# Peripheral aetiopathogenic drivers and mediators of Parkinson’s disease and co-morbidities: role of gastrointestinal microbiota

**DOI:** 10.1007/s13365-015-0357-8

**Published:** 2015-06-20

**Authors:** Sylvia M. Dobbs, R. John Dobbs, Clive Weller, André Charlett, Aisha Augustin, David Taylor, Mohammad A. A. Ibrahim, Ingvar Bjarnason

**Affiliations:** Pharmaceutical Sciences, King’s College London, London, UK; The Maudsley Hospital, London, UK; Department of Gastroenterology, King’s College Hospital, London, UK; Statistics Unit, National Infection Service, Public Health England, London, UK; Diagnostic Immunology Laboratory, King’s College and St Thomas’s Hospitals, London, UK

**Keywords:** Aetiology, Pathogenesis, Parkinson’s and overlap diseases, *Helicobacter*, Intestinal dysbiosis, Autoimmunity, Bystander damage

## Abstract

We seek an aetiopathogenic model for the spectrum of Parkinson’s disease (PD), functional bowel disease, depression and cognitive impairment. The adopted concept is that systemic immuno-inflammatory processes mediate neuro-inflammation. The model would be based on phenotype, exposome (including gastrointestinal microbiome), milieu (immuno-inflammatory and metabolome), human genetics and their interactions. It would enable a patient’s position, to be understood in terms of drivers, perpetuators and mediators, and a future position, with and without intervention, predicted. Even the cardinal facets of PD may have different drivers: halting one may allow escape down subordinate pathways. Peptic ulceration is prodromal to PD. In our randomised placebo-controlled trial, hypokinesia improved over the year following biopsy-proven *Helicobacter pylori* eradication and rigidity worsened. This was independent of any (stable, long *t*½) antiparkinsonian medication. There are pointers to an autoimmune process: for example, surveillance-confirmed hypokinesia effect was indication specific. During surveillance, successive antimicrobial courses, other than for *Helicobacter*, were associated with cumulative increase in rigidity. Exhibiting laxatives appeared to stem the overall temporal increase, despite antiparkinsonian medication, in rigidity. Thus, intestinal dysbiosis may be a major source of bystander neuronal damage. There are biological gradients of objective measures of PD facets on circulating inflammatory markers and leucocyte subset counts. Moreover, lactulose hydrogen breath test positivity for small-intestinal bacterial overgrowth (present in two thirds of PD patients) is associated with the same subsets: higher natural killer and total CD4+ counts and lower neutrophils. With greater aetiopathogenic understanding, relatively low cost and on-the-shelf medication could have a major impact. A new generation of animal models, based on the gut microbiome, is envisaged.

## Introduction

Since the shaking palsy, a rigid brady/hypokinetic syndrome with a characteristic tremor and stooped posture was described (Parkinson 1817); there have been few therapeutic milestones. Indeed, the only major advance, dopamine substitution therapy, dates back to description of dopamine deficiency in the basal ganglia (Ehringer and Hornykiewicz 1960).

Simultaneous co-morbidities, of which James Parkinson noted constipation, may have mediators, drivers and perpetuators in common. These include depression and mild cognitive impairment, with or without progression to dementia, as well as functional bowel disease. Unravelling the aetiopathogenesis, in a common disease with a long prodrome, will be jeopardised by not taking into account that individuals can be quantifiably down-the-pathway (Kirollos et al. 1993). A co-morbidity where the core condition is not overt may define an extreme of the spectrum and explain containment. Targeting aetiopathogenesis, rather than just phenotypic descriptors of disease, has the potential to open doors on cost-effective screening, prophylaxis, amelioration of the underlying processes and cure.

An aetiopathogenic model for this disease spectrum and its evolution with time and intervention is, thus, needed. Evolution refers both to change in individual facets of phenotype and shift within the spectrum. The model would enable a patient’s position in the spectrum to be understood in terms of drivers, perpetuators and mediators, and a future position, with and without intervention, predicted. Exposome, milieu (immuno-inflammatory and metabolome), human genetics and their interactions need to be considered as building blocks. Only ‘biomarkers’ which reflect driving or perpetuating forces can be useful in the modelling. Understanding the development of disease-specific pathophysiology requires longitudinal observational study to unmask associations, interventions to home in on cause/effect relationships and a new generation of animal models. A quantifiable aetiopathogenic model can be cross-referenced against quality of life and health economic outcomes.

The challenge requires (i) considering the whole disease spectrum; (ii) including pre-presentation states and attenuated or partial manifestations, not just ‘the tip of the iceberg’; (iii) assembling raw clinical clues without selectivity; (iv) using valid, sensitive, specific and reliable measures (objective where possible) of disease facets, which can track evolution; (v) stratifying paths of evolution into those intrinsic to initiation and the subsidiary; and (vi) animal models of the aetiopathogenesis that is not relying on surgical, chemical or genetic lesioning and so being downstream of, or out-with, environmental driving processes.

## Underpinning concepts and main ideas

Like other chronic diseases, Parkinson’s disease (PD) is multi-step and multi-factorial. Even more steps and factors, and biological gradients will be needed to explain co-morbidities and the spectrum of disease. Even the cardinal facets of PD may have different, not necessarily coincident, drivers: halting one may allow escape down subordinate pathways. However, all this does not preclude a systematic explanation.

The core concept is that neuro-inflammation in PD and overlap diseases is mediated by systemic immuno-inflammatory processes (Dobbs et al. 2008, 2012). It is not just reaction to aberrant protein deposition or degenerating neurons. There is indicative evidence that dysbiosis in the alimentary tract is the major driver of these processes (Augustin et al. 2014; Dobbs et al. 2008, 2010, 2012, 2013). Position of a patient within the disease spectrum is further determined by interaction with host genetics (risk and inflammatory), inflammatory and metabolic milieu and the exposome (including environmental factors, such as tobacco smoking). Intervention against drivers, perpetuators or mediators would allow disease modification.

## Some indicative models

### Influence of microbiome

Germ-free mice move more and take more risks: they have increased striatal synaptogenesis and dopamine/serotonin turnover (Bercik et al. 2011). In specific pathogen-free mice, non-absorbable antimicrobials increase exploratory behaviour and hippocampal brain-derived neurotrophic factor (BDNF). Gavage of caecal contents from a more outgoing mouse strain into a more timid increases both exploratory behaviour and BDNF, and vice versa. Specific probiotics cause behavioural change.

Important associations between stool bacteria microbiota and human health have been identified (Arumugam et al. 2011; Blottiere et al. 2013; Collins et al. 2012; Cotillard et al. 2013; Doré et al. 2013; Le Chatelier et al. 2013; Manichanh et al. 2006; Qin et al. 2014). There is evidence that stool microbial metagenomics can discriminate better for chronic disease than human genomics (Qin et al. 2014; Speliotes et al. 2010): interventions here should clarify cause/effect relationships. Psychiatric illness in irritable bowel syndrome (and inflammatory bowel disease) and autism spectrum disorder have been ascribed to dysbiosis (Doré et al. 2013). Autism recalls the restricted behaviour of PD. An inflammation-associated form of depression is described (Doré et al. 2013). Exhibition of the non-absorbable broad-spectrum antimicrobial rifaximin was accompanied by amelioration of parkinsonism associated with hepatic encephalopathy in three patients with cirrhosis and portosystemic shunting, in whom blood ammonia and electroencephalogram were unchanged (Kok et al. 2013). Imaging features in the globus pallidus, classical of parkinsonism in cirrhosis, were reduced. The only study of microbiota in PD is a cross-sectional comparison of 72 probands (almost all on antiparkinsonian medication) with 72 controls (with markedly increased frequency of cerebro- and cardio-vascular co-morbidities), without reference to dietary differences (Scheperjans et al. 2014).

### Influences on phenotype

#### Constipation and small-intestinal bacterial overgrowth

In PD, the frequency of defaecation diverges from that of controls three decades before the median age of neurological diagnosis (Charlett et al. 1997). Moreover, infrequent bowel movements are associated with a subsequent diagnosis (Abbott et al. 2001). Morphological and neurochemical changes of PD are found throughout the enteric nervous system and in dorsal vagal nuclei which serve the gastrointestinal tract (for review, see Dobbs et al. 2008).

Sixty-seven per cent of PD probands are lactulose hydrogen breath test (LHBT) positive for small-intestinal bacterial overgrowth (SIBO) on presentation (Dobbs et al. 2012). A likely cause is caeco-ileal bacterial reflux from an overloaded right colon. SIBO influences the immuno-inflammatory milieu, and biological gradients connect milieu to phenotype. Accompanying dysbiosis in the fermentation ‘bioreactor’ of the right colon may reduce production of anti-inflammatory substances such as short-chain fatty acids (Neish 2009).

#### Clues regarding rigidity

Surveillance of arm rigidity showed a significant temporal increase (7 % per year) in flexor rigidity, with consequent increase in the ratio, flexor to extensor rigidity, denoting simian posture (Augustin et al. 2014). Exhibiting laxatives, in general, was associated with stemming the increase, after adjustment for the various classes of long *t*½ antiparkinsonian medication (dopaminergic agonists, MAO-B inhibitors, amantadine, anticholinergics) and the limited use of levodopa in low dosage. Exhibiting the guanylate cyclase-C receptor agonist (linaclotide) was associated with reversing the temporal trend.

**Fig. 1:**
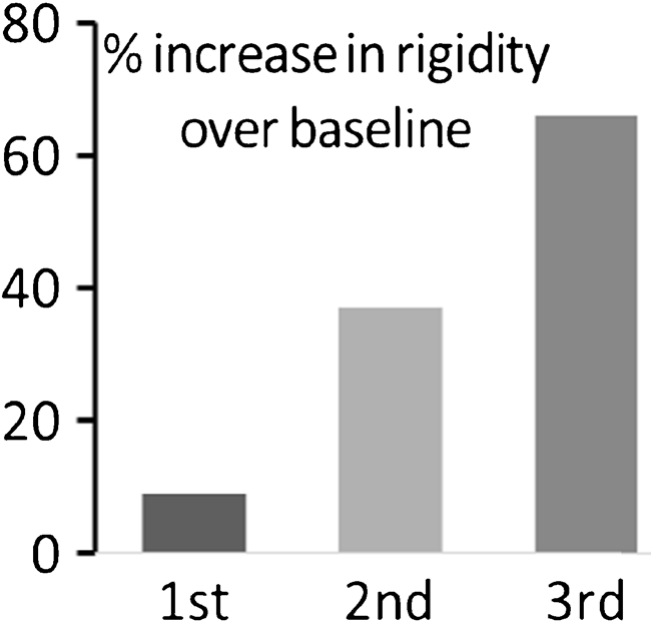
Cumulative worsening of objectively measured rigidity with successive antimicrobial interventions in PD

Successive courses of antimicrobials in PD are associated with cumulative increase in flexor rigidity (Fig. 1), over and above the effect of time and irrespective of indication (Dobbs et al. 2013).

#### Clues regarding brady/hypokinesia

**Fig. 2:**
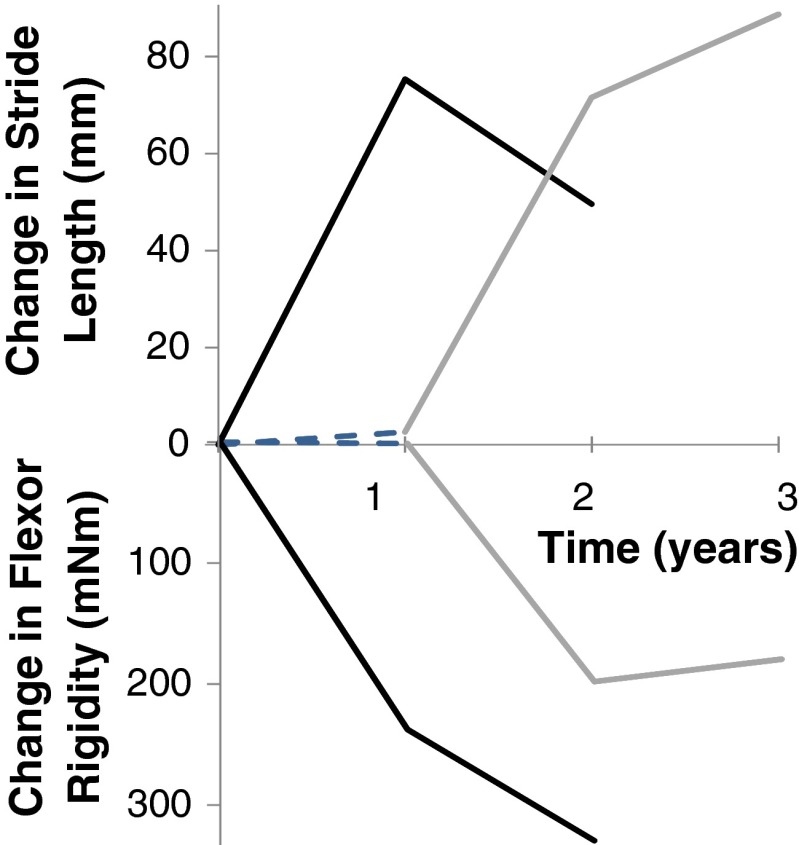
Schematic representation of the effect of *H. pylori* eradication on stride length and flexor rigidity in PD. Estimated mean time trends following successful blinded-active (*black*), open-active (*grey*) and placebo (*dashed*). Rejection of null hypothesis was based on double-blind protocol analysis of time trends in the primary outcome, stride length, reinforced by intention-to-treat analysis on its final measurement in blinded phase (*p =* 0.005), despite inclusion of the two proven eradication failures following blinded-active

That peptic ulceration is prodromal (Strang 1965) paved the way to exploring *Helicobacter pylori* in PD. In a randomised controlled trial (RCT), biopsy-proven *H. pylori* eradication reduced hypokinesia of gait in PD (Dobbs et al. 2010). Longitudinal observation showed indication specificity, in that antimicrobials for other indications did not improve hypokinesia (Dobbs et al. 2013). In the trial, whilst hypokinesia improved, rigidity worsened over the year post-eradication, both plateauing over the next (Fig. 2). There was overall clinical benefit. Improved hypokinesia was independent of any (stable, long *t*½) antiparkinsonian medication. (Receipt of levodopa was an exclusion.) Increased rigidity may flag acquisition of SIBO, since *H. pylori* and LHBT positivity are inversely related in PD (Dobbs et al. 2012). At present, the level of evidence is 1b since this is an individual RCT (OCEBM 2011).

In no disease, where *H. pylori* is causal, is it present in all cases. However, current or past *Helicobacter* infection may be a necessary though not sufficient player in developing the full syndrome. There is a lack of birth cohort effect for *H. pylori* in PD, as in gastric cancer and peptic ulcer where causal links with *H. pylori* are generally accepted (Dobbs et al. 2000). Danish population registers show increased prescription of anti-*Helicobacter* drugs in the 5 years prior to diagnosis (Nielsen et al. 2012). Dopaminergic agonists can prevent duodenal ulcer relapse in man (Sikiric et al. 1991), but whether by suppressing *H. pylori* is unknown.

#### Classical spousal approach to environmental causality

Spouses of PD probands are a short but highly significant ‘distance-down-the-pathway’ with respect to objective measures of PD facets (Kirollos et al. 1993, 1996; O’Neill et al. 1994; Weller et al. 1992). Probands and spouses have relative lymphopenia (with particular effect on B cells) (Charlett et al. 2009). There is a proportional increase in natural killer cell (NK) count in probands, in CD4+ in spouses. Half of the probands and a third of spouses have chronic functional bowel abnormality (Ellis et al. 2007). Like probands, two thirds of spouses are LHBT positive (Dobbs et al. 2012). The whole is difficult to attribute to selective mating or to learned or reactive behaviour. Neither is it explained by *H. pylori*: spouses had a lower frequency of *Helicobacter* anti-urease IgG enzyme-linked immunosorbent assay (ELISA) seropositivity than either probands or controls (Charlett et al. 2009), as though an acquired dysbiosis had suppressed it.

### Influence of immuno-inflammatory milieu

#### Biological gradients on circulating leukocyte subtypes

There are gradients of objective measures of facets of PD on blood leucocyte subtype counts (Dobbs et al. 2012). Brady/hypokinesia and flexor rigidity are worse the higher the NK count. Increased brady/hypokinesia was noted with *Helicobacter* positivity, over and above that explained by NK count and of a magnitude equivalent to that of a levodopa challenge. Association of rigidity with a higher NK count is modulated by the total CD4+ count. The CD4+ subset includes regulatory T cells (T-reg) which inhibit NK effector mechanisms. Tremor is worse with lower neutrophils: this may reflect neutrophil sequestration in the gut.

**Fig. 3:**
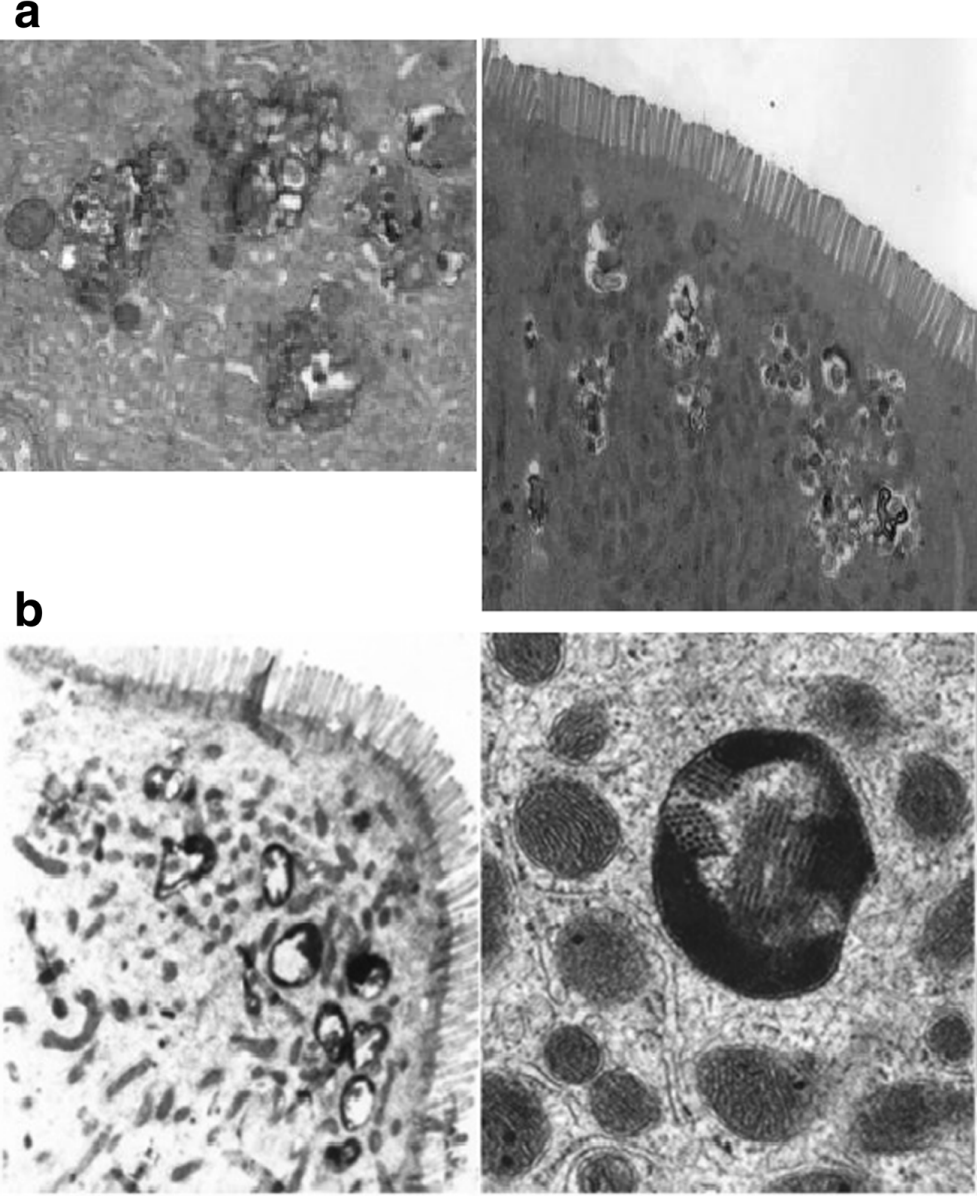
**a** Electron micrographs illustrating a cloud of irregular lysosomes in a duodenal enterocyte in a PD patient with SIBO, at low magnification (*right*) and higher (*left*). **b** Electron micrographs illustrating protein arrays encapsulated in a double membrane, at low magnification (*left*: multiple bodies) and higher (*right*: body amongst (normal) mitochondria), where arrays are seen longitudinally and in transverse section

LHBT positivity is associated with the same blood leucocyte subtypes: (higher) NK and CD4+ counts and (lower) neutrophils. Moreover, clouds of lysosomes seen in duodenal enterocytes in relation to luminal bacteria underline that SIBO is not an innocent bystander in PD (Fig. 3a) (Dobbs et al. 2012). The simplest biologically plausible explanation is that circulating leucocytes represent mediators of neuronal damage, and dysbiosis, flagged here by SIBO, represents a driver.

#### Biological gradients on circulating immune-inflammatory markers

There are gradients of objective measures of PD facets on serum cortisol and tumour necrosis factor alpha (TNF-α), and of global motor scores on peripheral blood mononuclear cell production of cytokines and nuclear factor-kappa B (NFκB) expression (Charlett et al. 1998; Dobbs et al. 1999; Reale et al. 2009). Cortisol is elevated by, on average, 17 % in PD. Serum interleukin-6 (IL-6) increases with age: it is elevated in PD by an amount equivalent to 10 years of ageing. Moreover, a higher concentration of IL-6, in blood collected 4 years previously, is predictive of incident PD (Chen et al. 2008).

Immuno-inflammatory activation can increase homocysteine production (Lazzerini et al. 2007). Hyperhomocysteinemia in PD (43 %) is explained, in small part, by the serum concentration of vitamin B_12_ (cobalamin), with no complementary effect of folate (Charlett et al. 2009). (Methyltetrahydrofolate acts as a methyl donor, and cobalamin is a co-factor, in remethylation of homocysteine to methionine by methionine synthetase.) Hyperhomocysteinemia is not explained by *Helicobacter* status or gastric atrophy. Impaired terminal ileal B_12_ absorption, associated with dysbiosis, might contribute. Although there was no evidence of frank B_12_ deficiency in PD, 16 % of probands had concentrations within the ‘equivocal range’ (Charlett et al. 2009). (Serum folate distribution was platykurtic.) Immuno-inflammatory activation may increase demand for B_12_ to such an extent that a concentration in the ‘equivocal’ range is pathological. Since SIBO both provokes an inflammatory response and increases bacterial utilisation of B_12_, it is likely to contribute to hyperhomocysteinemia in PD.

Hyperhomocysteinemia is associated with an increased risk of development of dementia and Alzheimer’s disease (AD) (Seshadri et al. 2002). Low and equivocal serum B_12_ concentrations, and the metabolically active fraction of serum cobalamin, have been implicated (Clarke et al. 1998; Refsum and Smith 2003; Seshadri et al. 2002), but the contribution of gastric atrophy and impaired ileal absorption is unknown. The low serum folate of AD (Clarke et al. 1998) appears to be an argument against SIBO (associated with increased synthesis of folate) being the main player, but demand for folate to detoxify homocysteine may be increased.

Using the western blot profile of IgG antibodies against electrophoretically separated *H. pylori* antigens, the predicted probability of being labelled as having PD was greatest with cytotoxin-associated gene product (CagA) positivity and vacuolating toxin negativity, and urease B negativity (Weller et al. 2005). With this pattern, the odds for having PD were increased fivefold at age 80 years. The predictive ability was not confined to those with current infection.

### Metabolome

From an aetiopathogenic standpoint, a key question, then, is whether the metabolome regulates systemic inflammation. In mice, short-chain fatty acids (SCFA), such as butyrate, produced by colonic bacterial fermentation, promote colonic T-reg cells and thereby suppress pro-inflammatory T cells (Smith et al. 2013). This suggests a mechanism for CD4+ modulation (T-reg component) of rigidity and fits with PD probands’ spouses having a proportional (protective) increase in total CD4+ count. However, whilst oral administration of SCFA to germ-free mice to address their deficit increased colonic T-regs, it had no effect on mesenteric lymph node, splenic or thymic T-regs. Human studies have linked fermentation products to behaviour (Collins et al. 2012). For example, high faecal propionic acid concentrations correlate with anxiety in irritable bowel syndrome.

Metabolomic interest in PD and AD has concentrated on markers of damage (e.g. hypoxia, oxidative stress and membrane lipid remodelling) in blood (Bogdanov et al. 2008; Orešič et al. 2011) and breath (Nakhleh et al. 2015).

### Bystander damage and cross-reactivity

The biological gradients described suggest bystander damage to the central nervous system in PD, driven by dysbiosis. Dysbiosis could account for the continuing substantia nigra microglial activation of PD (Pfeiffer 2009). This does not exclude added insults by intercurrent infection/its treatment.

In PD, nigral microglia express major histocompatibility antigens, including HLA-DR (McGeer et al. 1998). They secrete TNF-α, whilst dopaminergic neurons express its receptors and upregulate NFκB (Boka et al. 1994; Hunot et al. 1997). Nigral and cerebrospinal fluid (CSF) concentrations of other cytokines associated with innate immune response, IL-1β and IL-6, are elevated (Mogi et al. 1994, 1996). Although pro-inflammatory polymorphisms have not been identified as risks for PD in genome-wide studies, they might act as a conditional dependency of an effect modifier.

Adaptive immunity and autoimmunity may also have a role, separated in time from bystander damage or concurrent (Dobbs et al. 2008). Nigral dopaminergic neurons bind IgG, adjacent microglia express HLA-DR and high-affinity Fcγ antibody receptors, whilst peripheral lymphocytes are seen in relation to degenerating neurons (Orr et al. 2005). Serum, CSF and purified IgG from PD probands selectively inhibit dopamine uptake of rodent nigral dopaminergic neurons and selectively destroy neurons in vivo and in vitro (Chen et al. 1998; Dahlström et al. 1990; Defazio et al. 1994; McRae et al. 1986). Toxicity is dependent on complement and microglial Fcγ receptors (Defazio et al. 1994; He et al. 2002). Associations of PD with HLA-DR loci suggest classical autoimmunity (Ahmed et al. 2012; Hamza et al. 2010; International Parkinson Disease Genetics Consortium et al. 2011). Indeed, antinuclear antibody seropositivity flags poor response of hypokinesia to *H. pylori* eradication therapy (Dobbs et al. 2010). That *H. pylori* has been associated with hypokinesia when the infection load is low (detected by PCR only, not culture) is compatible with autoimmunity (Dobbs et al. 2008, 2010). There could also be cross-reactivity through innate pattern recognition of *Helicobacter* at the genus level (Dobbs et al. 2005) or of a broader microbial community.

The adaptive immune response in the substantia nigra in PD (Orr et al. 2005) and the presence of peripheral immune cells (as well as Lewy bodies) in therapeutically useful dopamine cell brain implants (Kordower et al. 2008) fit with a peripheral immune process driving neuronal damage (Lewy bodies are intracytoplasmic neuronal inclusions of misfolded α-synuclein, considered the gold standard for designation of PD). Indeed, in more advanced PD, the proportion of IgG-labelled nigral neurons decreases, but the activated microglia persist (Orr et al. 2005), suggesting that the immune-inflammatory process is driving neuronal damage, not vice versa. Elevated cortisol and cytokines in PD (Charlett et al. 1998; Dobbs et al. 1999) would increase permeability of the blood–brain barrier to peripheral immune cells, antigen, antibodies or products. There might also be vagal afferent signalling to microglia (Watkins 1995).

Outside the context of inflammation, effects of some probiotic bacteria on behaviour of experimental animals appear vagal dependent (Collins et al. 2012). Other workers (Bercik et al. 2011) have produced behavioural and neurochemical changes, apparently independent of the autonomic system and circulating cytokines, by manipulating the microbiota. They propose the pathway may involve neurally active substances (e.g. an antidepressant effect of butyrate).

### Abnormal mitochondria

Nigral and platelet mitochondrial dysfunction is described in PD (Dobbs et al. 2008). There is dysmorphology in duodenal enterocytes. Long, thin mitochondria, associated with the rough endoplasmic reticulum, are commonly seen, in the presence of SIBO but absence of recent *H. pylori* infection (Charlett et al. 2009). The complex branching also seen may result from failure to divide (Dobbs et al. 2008).

With current or recent *H. pylori* infection, arrays encapsulated in a double membrane are found in half of cases (Fig. 3b), lying among normal mitochondria (Dobbs et al. 2008; Ellis et al. 2007). There is a report of similar mitochondrial inclusions in cerebral neurons in Creutzfeldt–Jakob-like disease (Lewin and Edwards 1991). Alternatively, these bodies might be viroplasm, not mitochondria. Our electron microscopists (personal communication: Ellis D and Curry A) had not previously observed similar bodies but subsequently found examples in archived duodenal biopsies from two patients with human immunodeficiency virus (HIV) infection.

If these findings are replicated in enteric neurons and/or myocytes and are associated with mitochondrial dysfunction, they may provide a mechanism behind slow gastrointestinal transit. Indeed, cardiac and skeletal muscle mitochondrial hypofunction may contribute to hypotension and brady/hypokinesia.

## A viral primer as a supplementary explanation?

Faecal overload and SIBO are predisposed to by the slow GI transit of PD and may have detrimental feedback on it, but what initiates that slow transit? Could there be a viral primer? Enteroviruses infect via the gastrointestinal tract and are associated with neurological syndromes. Indeed, there is recent evidence of an enterovirus as a cause of encephalitis lethargica and post-encephalitic parkinsonism (Dourmashkin et al. 2012), and in our pilot study, using faecal samples taken at the start of a diarrhoeal episode, the frequency of enterovirus genogroup B RNA appeared high in PD patients and their spouses (personal communication: Appleton H).

A viral primer could also be involved in the relative lymphopenia seen in both PD patients and their spouses, compared with controls. This robust finding was not explained by antiparkinsonian medication, *Helicobacter* status or breath hydrogen. A relatively benign retrovirus might explain this and the slow transit. The comparatively high frequency of *Dientamoeba fragilis* (18 % of PD probands and their spouses cf*.* 2.6 % of routine parasitology requests) (Ellis et al. 2007) could flag mild acquired immunodeficiency. The epidemiology of IP and HIV is distinct, but parkinsonism is seen in uncomplicated HIV infection (as well as with opportunistic infections in acquired immunodeficiency syndrome) (Koutsilieri et al. 2002; Karlsen et al. 1992; Berger and Arendt 2000). Moreover, jejunal autonomic denervation is described with HIV infection (Dourmashkin et al. 2012). Although Lewy bodies are not reported in HIV (Koutsilieri et al. 2002), motor dysfunction compatible with basal ganglia damage is found in early and basal ganglia dopaminergic cell loss is seen without clinical parkinsonism. In simian immunodeficiency virus-infected monkeys, nigrostriatal dopamine is halved within 2 months (Koutsilieri et al. 2002).

## Time sequence: a process initiated by, driven from, the gastrointestinal tract?

The time sequence of constipation and peptic ulceration fits with the misfolded protein theory of PD pathogenesis. Aggregates of misfolded α-synuclein are found throughout the enteric nervous system and in dorsal vagal nuclei (Braak et al. 2006). A subpopulation of α-synuclein-expressing myenteric neurons, synaptically connected to vagal efferents, has been described in rodents (Phillips et al. 2008). As PD evolves, aggregation spreads from the brainstem to the substantia nigra, areas of the midbrain and basal forebrain, eventually reaching the neocortex (Braak et al. 2003).

Although misfolded proteins can aggregate by template replication in a prion-like manner (Luk et al. 2009), it is not known what initiates or ‘seeds’ misfolding, converts containment to progression or drives progression. Enteric α-synuclein aggregates are described with local inflammation in the gut, both clinical and experimental colitis (Grathwohl et al. 2013). In early PD, inflammation, measured by imaging microglial activation, in the affected nigrostriatal pathway accompanies loss of presynaptic dopamine transporter (Ouchi et al. 2005) and becomes more widespread on follow-up (Ouchi et al. 2009). Central neuro-inflammation may have peripheral mediators (see ‘Influence of immuno-inflammatory milieu’).

Effective therapeutic strategies to prevent, contain and clear α-synuclein deposition may be jeopardised by a persistent driver presented by gut microbiota or by its immuno-inflammatory or metabolomic mediator(s).

## Effecting a paradigm shift

‘The best science often emerges from situations where results carefully obtained do not fit within accepted paradigms’ (Prusiner 1982). Whereas an array of stances is legitimate in the humanities, in science, a paradigm shift (Kuhn 1970) is all-or-nothing. It may rest on a single discovery, or, as here, unify a network of novel findings. Practicality can be as important as explanation in effecting a shift. The hypothesis that *H. pylori* is the usual cause of peptic ulcer (Marshall and Warren 1984) was dismissed until it became evident that eliminating *Helicobacter* effected cure. Until then, the success of H_2_ receptor antagonists in healing an ulcer had made the new paradigm unnecessary. Although requiring no change in disciplinary ownership, just in focus, from modifying gastric physiology to antimicrobial therapy, shifting that paradigm took more than a decade. In our current example, there is a need for new disciplinary interfaces in order to yield disease-modifying therapies. Considering PD only in terms of progressive, self-perpetuating, degeneration relegates any environmental influence to being, at best, remote hit-and-run. Nosological classification as ‘non-communicable’ (WHO) reinforces this. Regarding any systemic illness in PD as an ‘intercurrent event’, as opposed to co-morbidity, compounds the problem.

What is sure, in a disease peculiar to man, is that re-evaluating the patient is a good starting point. Detective work is needed, where subtle clues are uncovered and statistical analysis builds on meticulous clinical observation. In such exploratory studies, it is necessary to understand what is measured and what influences it, explore effect modification, examine biological plausibility and seek corroborative evidence. Not until a large number of clues have been assimilated will their position within a causal scheme become more certain. Pragmatic studies can then be conducted for the testable cause/effect hypotheses generated. This is the antithesis of one-step pragmatism. A scientifically challenging causal pathway does not preclude a clinical solution sufficiently simple to be assimilated into practice.

Opposition in principle is the great delayer. ‘Student’ of the *t* test wrote of RA Fisher’s concise statistical/mathematical text ‘When I came to “evidently” I know that it means two hours hard work at least before I can see why’ (Bodmer 2003). Getting over ‘why not’ in the face of inertia of consensus opinion is far more time consuming. ‘Consensus is always conditioned by the antecedent knowledge and its interpretation, and hence is time dependent….it should be associated with permanent criticism, which hopefully will induce corrective changes’ (Vonka 2000). Grassroot opinion, from people with the chronic disease who want a cure, is important in influencing professional consensus.

## Conclusion

Biological plausibility of the underpinning concept and main ideas lies in the fit of a constellation of observational and intervention studies. In particular, biological gradients (Hill 1965) of repeated (antimicrobial) interventions and circulating inflammatory markers on measures of facets of PD add to the RCT evidence for the gastrointestinal microbiome being involved in its causality. Metagenomics can be used to address the Bradford Hill predicament of although ‘The clear dose–response curve admits of a simple explanation’ (causality), ‘Often the difficulty is to secure some satisfactory quantitative measure of the environment which will permit us explore this dose–response.’

It is envisaged that PD will be reclassified as a systemic condition in response to immuno-inflammatory activation, influenced by microbiota, tempered by human genetics, within a spectrum of neuropsychiatric and gastroenterological conditions. Dichotomous classification of PD is misconceived. Since facets may have different, non-coincident, driving forces, defining phenotype by their objective quantification is a sine qua non for progress. Lumping facets together (global clinical scores) presumes they progress in parallel within-subject, in set proportion between, and share driving forces.

A ‘depth-in-breadth’ approach maximises the potential of yielding new targets, such as optimising microbiota, eradicating pathogens, immune modulation (including cross-talk at the mucosal level and signalling to/activity of local, systemic and brain immune system) and optimising the metabolome. Relevant animal models, driven by gastrointestinal microbiome and autoimmunity, would consolidate this approach. Elucidating the cause of slow transit and relative lymphopenia may reveal a trigger event. The potential is as a catalyst for change in approach to chronic disease in general, with major social, health and financial implications.

Where drivers, perpetuators and mediators remain active, attempts at neuronal replacement, repair and regeneration and symptomatic treatment may underperform. A complex causal pathway does not preclude interim clinical solutions.
